# Mediterranean diet adherence and risk of esophageal and gastric cancer subtypes in the Netherlands Cohort Study

**DOI:** 10.1007/s10120-019-00927-x

**Published:** 2019-02-15

**Authors:** Maya Schulpen, Petra H. Peeters, Piet A. van den Brandt

**Affiliations:** 1 Department of Epidemiology, GROW-School for Oncology and Developmental Biology, Maastricht University Medical Centre, P.O. Box 616, 6200 MD Maastricht, The Netherlands; 2 Julius Center for Health Sciences and Primary Care, University Medical Center Utrecht, Utrecht, The Netherlands; 3 Department of Epidemiology, CAPHRI-School for Public Health and Primary Care, Maastricht University Medical Centre, Maastricht, The Netherlands

**Keywords:** Mediterranean diet, Esophageal neoplasms, Stomach neoplasms, Cohort studies, Epidemiology

## Abstract

**Background:**

Mediterranean diet (MD) adherence has been associated with reduced risks of esophageal and gastric cancer (subtypes) in a limited number of studies. We prospectively investigated associations between MD adherence and risks of esophageal squamous cell carcinoma (ESCC), esophageal adenocarcinoma (EAC), gastric cardia adenocarcinoma (GCA), and gastric non-cardia adenocarcinoma (GNCA) in a Dutch cohort.

**Methods:**

Analyses were conducted using data from the 120852 participants of the Netherlands Cohort Study (NLCS), who were aged between 55 and 69 years at enrollment. Various MD scores, with and without alcohol, were calculated to estimate MD adherence. Using 20.3 years of follow-up, 133 ESCC, 200 EAC, 191 GCA, and 586 GNCA cases could be included in multivariable Cox regression analyses.

**Results:**

Of the investigated scores, the alternate Mediterranean diet score without alcohol (aMEDr) performed best. aMEDr was inversely associated with risks of GCA and GNCA in men and women. However, statistical significance was only reached in men [*p*_trend_: 0.019 (GCA), 0.016 (GNCA)]. Furthermore, higher aMEDr values were significantly associated with a reduced ESCC risk in men [HR_per two−point increment_ (95% CI) = 0.57 (0.41–0.80), *p*_trend_ = 0.013], but not in women (*p*_heterogeneity_ = 0.008). There was no evidence of an association between aMEDr and EAC risk. Educational level was a significant effect modifier for the association between aMEDr and GNCA risk (*p*_heterogeneity_ = 0.0073).

**Conclusions:**

Higher MD adherence was associated with reduced risks of ESCC, GCA, and GNCA in the NLCS. However, the decreased ESCC risk might be limited to men.

**Electronic supplementary material:**

The online version of this article (10.1007/s10120-019-00927-x) contains supplementary material, which is available to authorized users.

## Introduction

Cancers of the esophagus (sixth place) and stomach (third place) were amongst the most common causes of cancer-related death in the world in 2012 [1]. Two histologic types of esophageal cancer can be distinguished, namely esophageal squamous cell carcinoma (ESCC) and esophageal adenocarcinoma (EAC) [2]. Based on anatomic location, gastric cancers are subdivided into gastric cardia adenocarcinomas (GCA) and gastric non-cardia adenocarcinomas (GNCA) [3]. Different etiologies have been suggested for these subtypes [2, 3]. In the past decades, incidence rates of EAC and GCA have been rising in many European countries and the United States (US) [4, 5].

The traditional Mediterranean diet (MD) is characterized by a high consumption of vegetables, fruits, whole grains, and other plant foods, with olive oil as the principal source of fat. Foods from animal origin are consumed in low amounts in the MD, whereas alcohol intake is moderate [6–8]. The relation between *a priori* defined MD adherence and the incidence of esophageal and/or gastric cancer (subtypes) has been the topic of a limited number of studies [9–13]. In these studies, higher MD adherence has been associated with reduced risks of ESCC, GCA, GNCA, and total gastric cancer (GC), but results were not always significant and sometimes inconsistent, primarily with respect to the gastric cancer subtypes [9–13].

Information bias due to reversed causation is a major concern when investigating relations between dietary factors and gastrointestinal cancer risk, because preclinical disease symptoms may cause patients with gastrointestinal tumors to alter their dietary habits already before clinical diagnosis. Another concern is recall bias, which could particularly be a problem in case–control studies. For these reasons, the effects of dietary factors on gastrointestinal cancers should be investigated prospectively, if possible. So far, associations between MD adherence and risks of esophageal and/or gastric cancer subtypes have been prospectively investigated in only two cohort studies [10, 11]. Therefore, more prospective evidence on this topic is desired.

This study prospectively investigated the association of MD adherence with the risk of esophageal and gastric cancer subtypes (ESCC, EAC, GCA, and GNCA) in the Netherlands Cohort Study (NLCS). We assessed MD adherence using various *a priori* defined MD scores, with and without alcohol, and examined associations for men and women separately.

## Methods

### Study population and cancer follow-up

The NLCS is a Dutch population-based cohort study, which has been described in detail previously [14–17]. In summary, the NLCS comprises 58279 men and 62573 women, aged 55–69 years, from 204 Dutch municipalities, who completed a self-administered questionnaire on diet and other cancer risk factors at baseline in September 1986. A case-cohort design was used to allow for efficient processing and analysis of the data. Therefore, a subcohort (*N* = 5000) was randomly sampled immediately after baseline to estimate the number of person-years at risk. Cases were obtained from the total cohort. Vital status information for subcohort members was acquired biennially using municipal population registries [14, 17, 18]. Approval for the NLCS was obtained from the institutional review boards of Maastricht University and the Netherlands Organization for Applied Scientific Research. All cohort members agreed to participate by filling out the questionnaire.

**Fig. 1:**
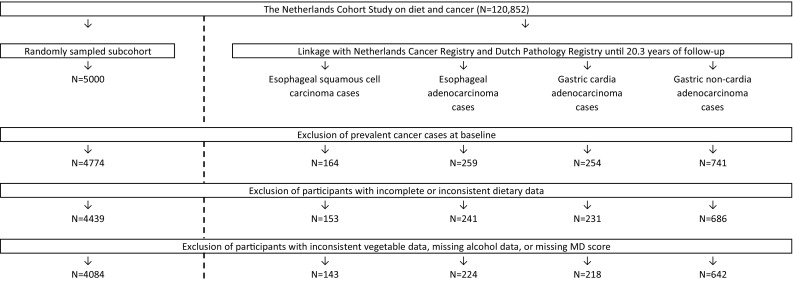
Flow diagram of the number of NLCS participants, who are eligible for inclusion in the analyses (case-cohort design). *N* number of subjects, *MD* Mediterranean diet, *NLCS* Netherlands Cohort Study

Follow-up for cancer incidence was carried out annually by record linkage with the Netherlands Cancer Registry and the Dutch Pathology Registry (PALGA) [15]. The NLCS cohort was followed-up for 20.3 years until December 31st 2006. To be eligible for inclusion in the present study, esophageal and gastric cancer cases had to be incident and microscopically confirmed with known tumor histology and topography. Based on the International Classification of Diseases for Oncology, Third Edition, esophageal and gastric cancers were classified into ESCC (C15, histology codes: 8050–8076), EAC (C15, histology codes: 8140, 8141, 8190–8231, 8260–8263, 8310, 8430, 8480–8490, 8560 and 8570–8572), GCA (C16.0), and GNCA (C16.1–C16.9). Subjects were excluded if they had prevalent cancer at baseline (except any type of skin cancer) and/or incomplete or inconsistent data on diet, alcohol, or MD adherence. In total, 143 ESCC, 224 EAC, 218 GCA, and 642 GNCA cases and 4084 subcohort members could be included in the analyses (Fig. 1).

### Exposure assessment

A self-administered, 150-item, semi-quantitative food frequency questionnaire (FFQ) was utilized to assess the participant’s habitual diet during the year preceding baseline. Validity and reproducibility of this FFQ have been described previously [16, 19]. Mean daily nutrient intakes were calculated using the Dutch food composition table of the year 1986 [20].

### Mediterranean diet adherence

MD adherence was measured using two variants of the traditional Mediterranean diet score (tMED) created by Trichopoulou et al., namely the alternate Mediterranean diet score (aMED) and the modified Mediterranean diet score (mMED) [21–25]. Differences in daily energy intakes were taken into account in the calculation of the MD scores by standardizing daily food intakes to 2000 (women) and 2500 (men) kilocalories [21, 25]. aMED assesses relative MD adherence based on the mean daily intakes of nine dietary components [24, 25]. Each component is scored by 0 or 1 points with the maximum score of 9 representing the highest level of MD adherence. Subjects receive 1 point for intakes at or above the sex-specific median of vegetables (excluding potatoes), legumes, fruits, nuts, whole grains, fish, and the ratio of monounsaturated to saturated fatty acids (MUFA:SFA ratio). The intake of red and processed meats is scored inversely. In addition, 1 point is assigned to a moderate alcohol intake [5–25 grams per day (g/day) for both sexes] [24, 25]. mMED is calculated in a similar way as aMED, but includes slightly different dietary components [23]. In mMED, intakes of fruits and nuts are grouped together, and total cereal and meat intake is scored. In addition, 1 point is obtained for a dairy intake below the sex-specific median. To improve the usage of mMED in non-Mediterranean populations, the fatty acid quality is measured by the ratio of unsaturated fatty acids (MUFA + polyunsaturated fatty acids) to SFA. Finally, a moderate alcohol intake is defined differently for men (10–50 g/day) and women (5–25 g/day) [23]. (Heavy) alcohol consumption has been associated with an increased risk of ESCC and probably GC [26]. Therefore, MD adherence was also assessed using aMED and mMED variants without alcohol (aMEDr and mMEDr, respectively), which had maximum scores of 8 points.

### Statistical analyses

All analyses were performed separately for men and women, unless otherwise specified. Hazard ratios (HRs) and 95% confidence intervals (95% CIs) for the associations of MD adherence with incidence of esophageal and gastric cancer subtypes were estimated using Cox proportional hazards models with follow-up duration as time variable. Person-years at risk for subcohort members were calculated from baseline until the diagnosis of esophageal or gastric cancer, death, emigration, loss to follow-up, or end of follow-up, whichever came first. Standard errors were estimated using the Huber–White sandwich estimator to account for the increased variance because of subcohort sampling [27]. To verify that all variables met the proportional hazards assumption, scaled Schoenfeld residuals tests and –ln(–ln) survival plots were used [28]. A time-varying covariate was included in the model when a potential confounder violated the proportional hazards assumption and inclusion of a time-varying covariate altered the HR of the MD score.

MD scores were included as categorical [low: 0–3, middle: 4–5, high: 6–8(9)] and continuous (per two-point increment) terms in age-adjusted and fully adjusted analyses [23, 25]. Tests for trends were performed by assigning sex-specific median values among subcohort members to the MD score categories and fitting these as continuous terms in the Cox regression models. To correct for potential confounding, the following set of literature-selected variables was included in fully adjusted Cox models: age at baseline, sex (except for sex-specific models), cigarette smoking status, cigarette smoking frequency, cigarette smoking duration, body mass index (BMI), total daily energy intake, alcohol consumption (except for models containing original MD scores including alcohol), highest level of education, non-occupational physical activity, and family history of esophageal cancer (for ESCC and EAC) or gastric cancer (for GCA and GNCA).

Akaike’s Information Criterion (AIC) was used to compare the performances of models containing aMEDr and mMEDr [29]. Furthermore, it was evaluated if inclusion of alcohol in the MD scores affected the model fits. Considering that (heavy) alcohol consumption has been associated with an increased risk of ESCC and probably GC, MD scores without alcohol are prioritized in the results section of this article and subsequent analyses were only performed using aMEDr [26]. Moreover, we prefer the use of aMEDr to assess MD adherence, because aMEDr-containing models had similar or better performances than mMEDr-containing models in the NLCS, both in the present and earlier analyses [30, 31].

The relative importance of the individual aMEDr components was investigated in two ways. First, all aMEDr components were entered simultaneously as dichotomous variables into fully adjusted Cox models. Second, HRs per two-point increment were estimated upon alternate removal of each aMEDr component from the sum score, one at a time, using the method described by Trichopoulou et al. [32]. In addition, analyses stratified by cigarette smoking status, alcohol consumption, BMI, and educational level were performed. The statistical significance of possible differences across strata was tested by including interaction terms between aMEDr and the potential effect modifiers. Finally, sensitivity analyses were performed in which the first 2 years of follow-up were excluded. Men and women were combined in the stratified and sensitivity analyses to increase the power. All statistical analyses were conducted using Stata version 15 (StataCorp LLC, College Station, TX, USA). Reported *p* values are two-sided and *p* values below 0.05 were considered statistically significant.

## Results

Sex-specific baseline characteristics of cases and subcohort members are presented in Table 1. Male ESCC, GCA, and GNCA cases had lower MD adherence than subcohort members. No clear differences in MD adherence were observed between female cases and subcohort members. Concerning potential confounding factors, cases were older (except for male ESCC and EAC cases) and less often never smokers (except for female EAC cases) than subcohort members. In addition, alcohol consumption was higher in ESCC and GCA (men only) cases, but lower in EAC cases (women only). Finally, the mean BMI was lower in ESCC cases, but higher in EAC and GCA cases.

**Table 1:** Baseline characteristics of the NLCS subcohort, ESCC, EAC, GCA, and GNCA cases

	Men										Women									
	NLCS subcohort		Cases								NLCS subcohort		Cases							
	*N* = 2057		ESCC (*N* = 84)		EAC (*N* = 179)		GCA (*N* = 184)		GNCA (*N* = 436)		*N* = 2027		ESCC (*N* = 59)		EAC (*N* = 45)		GCA (*N* = 34)		GNCA (*N* = 206)	
aMEDr	3.9	(1.6)	3.2	(1.5)	4.0	(1.6)	3.7	(1.5)	3.6	(1.5)	4.0	(1.6)	4.0	(1.5)	3.9	(1.5)	3.8	(1.7)	3.7	(1.7)
mMEDr	4.0	(1.5)	3.6	(1.4)	4.0	(1.5)	3.8	(1.5)	3.9	(1.4)	4.0	(1.5)	4.2	(1.5)	3.8	(1.5)	4.1	(1.4)	4.0	(1.5)
Age (years)^a^	61	(7)	61	(7)	61	(6)	62	(7)	62	(7)	61	(7)	63	(6)	62	(7)	62	(7)	62	(7)
Never cigarette smokers (%)	12.8		9.5		8.4		8.7		9.4		57.7		35.6		62.2		47.1		51.5	
Cigarette smoking frequency (cig/day)^ab^	15	(10)	20	(5)	20	(15)	15	(15)	15	(10)	10	(13)	15	(10)	10	(14)	10	(15)	10	(15)
Cigarette smoking duration (years)^ab^	36	(17)	40	(15)	35	(17)	37	(18)	40	(15)	30	(20)	39	(15)	35	(16)	29	(25)	30	(20)
Higher vocational education or university (%)	19.3		21.7		19.9		19.0		12.0		9.5		5.1		6.7		14.7		6.4	
Alcohol consumption (g/day)^a^	9.7	(20.9)	24.2	(29.8)	9.3	(23.6)	12.4	(21.6)	10.0	(21.9)	1.6	(7.8)	5.9	(20.7)	1.2	(12.4)	1.6	(5.4)	1.5	(8.2)
Daily energy intake (kcal)	2162	(501)	2057	(453)	2163	(474)	2132	(563)	2190	(504)	1687	(392)	1755	(384)	1631	(410)	1813	(338)	1666	(386)
Body mass index (kg/m^2^)	24.9	(2.6)	24.5	(3.2)	25.6	(2.5)	25.5	(2.6)	24.8	(2.8)	25.0	(3.5)	24.0	(3.6)	26.9	(4.3)	26.5	(4.3)	25.3	(3.9)
Non-occupational physical activity > 60 min/day (%)	50.7		41.5		53.6		51.4		51.5		44.3		37.3		42.2		41.2		39.0	
Family history of esophageal cancer (%)	0.7		0.0		1.1		1.1		1.2		0.9		3.4		6.7		0.0		1.5	
Family history of gastric cancer (%)	6.8		9.5		7.3		9.2		11.2		6.7		5.1		6.7		5.9		11.2	

The % missing values in the NLCS subcohort was < 5% for all variables included in this table, with the exception of cigarette smoking frequency (5.7%) in men. Mean (SD) values are reported unless otherwise specified

*NLCS* Netherlands cohort study, *ESCC* esophageal squamous cell carcinoma, *EAC* esophageal adenocarcinoma, *GCA* gastric cardia adenocarcinoma, *GNCA* gastric non-cardia adenocarcinoma, *N* number of subjects, *aMEDr* alternate Mediterranean diet score without the alcohol component, *mMEDr* modified Mediterranean diet score without the alcohol component, *cig*/*day* cigarettes per day, *g*/*day* grams per day, *kcal* kilocalories, *kg* kilogram, *m* meter, *min*/*day* minutes per day, *SD* standard deviation, *IQR* interquartile range

^a^Median (IQR) values are reported

^b^Median (IQR) values for frequency and duration of smoking were based on the former and current smokers

Tables 2 (men) and 3 (women) show fully adjusted associations between MD adherence, assessed using various MD scores, and the risk of esophageal and gastric cancer subtypes. Not all eligible study participants could be included in the Cox models because of missing values in covariates. Results of the age-adjusted analyses are presented in Online Resource 1.

**Table 2:** Fully adjusted associations of aMED and mMED (including and excluding alcohol) with the risk of esophageal and gastric cancer subtypes in male NLCS participants

	PY_subcohort_	ESCC		EAC		GCA		GNCA	
		Cases	HR (95% CI)^a^	Cases	HR (95% CI)^a^	Cases	HR (95% CI)^a^	Cases	HR (95% CI)^a^
aMEDr									
0–3	11889	46	1.00	59	1.00	71	1.00	190	1.00
4–5	12569	25	0.56 (0.33–0.94)	70	1.18 (0.81–1.72)	74	1.00 (0.71–1.41)	155	0.79 (0.62–1.01)
6–8	4792	5	0.35 (0.14–0.89)	28	1.33 (0.81–2.18)	13	0.48 (0.26–0.89)	45	0.65 (0.45–0.94)
*P*_trend_			0.013		0.248		0.019		0.016
Continuous, per 2 pts	29250	76	0.57 (0.41–0.80)	157	1.19 (0.96–1.47)	158	0.86 (0.70–1.06)	390	0.82 (0.71–0.95)
aMED^b^									
0–3	9233	38	1.00	46	1.00	57	1.00	153	1.00
4–5	13035	29	0.61 (0.36–1.01)	71	1.15 (0.77–1.72)	71	0.91 (0.63–1.31)	166	0.78 (0.61–1.01)
6–9	6983	9	0.38 (0.18–0.80)	40	1.28 (0.81–2.02)	30	0.74 (0.46–1.18)	71	0.68 (0.49–0.93)
*P*_trend_			0.004		0.291		0.241		0.011
Continuous, per 2 pts	29250	76	0.62 (0.47–0.83)	157	1.11 (0.91–1.37)	158	0.90 (0.74–1.10)	390	0.85 (0.74–0.98)
mMEDr									
0–3	10920	39	1.00	61	1.00	66	1.00	156	1.00
4–5	13549	29	0.57 (0.32–0.99)	68	0.93 (0.64–1.36)	75	0.92 (0.64–1.31)	182	0.92 (0.72–1.18)
6–8	4782	8	0.53 (0.24–1.19)	28	1.07 (0.66–1.73)	17	0.57 (0.33–1.00)	52	0.78 (0.55–1.12)
*P*_trend_			0.097		0.791		0.047		0.174
Continuous, per 2 pts	29250	76	0.65 (0.46–0.90)	157	1.04 (0.82–1.31)	158	0.82 (0.66–1.03)	390	0.93 (0.80–1.09)
mMED^b^									
0–3	7782	25	1.00	45	1.00	50	1.00	109	1.00
4–5	14091	39	0.87 (0.51–1.51)	69	0.82 (0.54–1.22)	72	0.75 (0.51–1.11)	199	0.95 (0.73–1.25)
6–9	7377	12	0.52 (0.26–1.07)	43	1.02 (0.65–1.60)	36	0.73 (0.46–1.16)	82	0.78 (0.56–1.08)
*P*_trend_			0.065		0.725		0.262		0.111
Continuous, per 2 pts	29250	76	0.78 (0.58–1.04)	157	1.03 (0.82–1.30)	158	0.86 (0.70–1.07)	390	0.93 (0.81–1.08)

*aMED* alternate Mediterranean diet score, *mMED* modified Mediterranean diet score, *NLCS* Netherlands Cohort Study, *ESCC* esophageal squamous cell carcinoma, *EAC* esophageal adenocarcinoma, *GCA* gastric cardia adenocarcinoma, *GNCA* gastric non-cardia adenocarcinoma, *PYsubcohort* person-years in the subcohort, *HR* hazard ratio, *CI* confidence interval, *aMEDr* alternate Mediterranean diet score without the alcohol component, *pts* points, *mMEDr* modified Mediterranean diet score without the alcohol component

^a^Adjusted for age at baseline (years), cigarette smoking status (never, former, current), cigarette smoking frequency (cigarettes smoked per day, centered), cigarette smoking duration (years, centered), body mass index (kilograms per meter^2^), daily energy intake (kilocalories), alcohol consumption (grams per day), highest level of education (primary school or lower vocational, secondary school or medium vocational, higher vocational or university), non-occupational physical activity (≤ 30, > 30-≤60, > 60-≤90, > 90 min per day), family history of esophageal cancer (for esophageal cancer subtypes; no, yes), and family history of gastric cancer (for gastric cancer subtypes; no, yes)

^b^Not adjusted for alcohol consumption

**Table 3:** Fully adjusted associations of aMED and mMED (including and excluding alcohol) with the risk of esophageal and gastric cancer subtypes in female NLCS participants

	PY_subcohort_	ESCC		EAC		GCA		GNCA	
		Cases	HR (95% CI)^a^	Cases	HR (95% CI)^a^	Cases	HR (95% CI)^a^	Cases	HR (95% CI)^a^
aMEDr									
0–3	12254	21	1.00	19	1.00	15	1.00	92	1.00
4–5	15123	27	1.18 (0.62–2.25)	18	0.80 (0.42–1.54)	12	0.65 (0.30–1.42)	71	0.70 (0.50–0.98)
6–8	6278	9	1.13 (0.48–2.66)	6	0.71 (0.27–1.88)	6	0.85 (0.33–2.18)	33	0.85 (0.56–1.31)
*P*_trend_			0.756		0.480		0.726		0.410
Continuous, per 2 pts	33655	57	1.09 (0.76–1.57)	43	0.98 (0.67–1.44)	33	0.82 (0.51–1.33)	196	0.83 (0.67–1.01)
aMED^b^									
0–3	10734	18	1.00	15	1.00	14	1.00	84	1.00
4–5	14697	24	1.10 (0.56–2.15)	19	1.00 (0.50–1.99)	11	0.54 (0.24–1.24)	73	0.69 (0.49–0.97)
6–9	8224	15	1.41 (0.65–3.03)	9	0.92 (0.38–2.21)	8	0.75 (0.30–1.91)	39	0.73 (0.49–1.10)
*P*_trend_			0.373		0.851		0.653		0.153
Continuous, per 2 pts	33655	57	1.09 (0.77–1.53)	43	1.05 (0.73–1.51)	33	0.80 (0.51–1.27)	196	0.83 (0.69–1.01)
mMEDr									
0–3	11675	17	1.00	17	1.00	9	1.00	75	1.00
4–5	16510	31	1.58 (0.83–3.03)	19	0.75 (0.37–1.54)	18	1.43 (0.63–3.27)	91	0.92 (0.66–1.28)
6–8	5470	9	1.62 (0.68–3.87)	7	0.89 (0.35–2.26)	6	1.39 (0.49–3.94)	30	0.95 (0.60–1.50)
*P*_trend_			0.231		0.793		0.538		0.797
Continuous, per 2 pts	33655	57	1.33 (0.89–2.00)	43	0.89 (0.58–1.36)	33	1.03 (0.66–1.61)	196	0.98 (0.80–1.21)
mMED^b^									
0–3	9693	15	1.00	16	1.00	9	1.00	63	1.00
4–5	16579	30	1.34 (0.68–2.66)	17	0.59 (0.29–1.22)	14	0.86 (0.36–2.05)	98	0.95 (0.67–1.33)
6–9	7383	12	1.35 (0.58–3.10)	10	0.83 (0.36–1.92)	10	1.37 (0.53–3.51)	35	0.80 (0.51–1.26)
*P*_trend_			0.511		0.793		0.429		0.327
Continuous, per 2 pts	33655	57	1.28 (0.89–1.83)	43	0.97 (0.65–1.45)	33	0.99 (0.64–1.54)	196	0.97 (0.79–1.18)

*aMED* alternate Mediterranean diet score, *mMED* modified Mediterranean diet score, *NLCS* Netherlands Cohort Study, *ESCC* esophageal squamous cell carcinoma, *EAC* esophageal adenocarcinoma, *GCA* gastric cardia adenocarcinoma, *GNCA* gastric non-cardia adenocarcinoma, *PYsubcohort* person-years in the subcohort, *HR* hazard ratio, *CI* confidence interval, *aMEDr* alternate Mediterranean diet score without the alcohol component, *pts* points, *mMEDr* modified Mediterranean diet score without the alcohol component

^a^Adjusted for age at baseline (years), cigarette smoking status (never, former, current), cigarette smoking frequency (cigarettes smoked per day, centered), cigarette smoking duration (years, centered), body mass index (kilograms per meter^2^), daily energy intake (kilocalories), alcohol consumption (grams per day), highest level of education (primary school or lower vocational, secondary school or medium vocational, higher vocational or university), non-occupational physical activity (≤ 30, > 30-≤60, > 60-≤90, > 90 min per day), family history of esophageal cancer (for esophageal cancer subtypes; no, yes) and family history of gastric cancer (for gastric cancer subtypes; no, yes)

^b^Not adjusted for alcohol consumption

High MD adherence according to aMEDr was associated with significantly reduced risks of ESCC, GCA, and GNCA in men [HR_high vs. low_ (95% CI): ESCC = 0.35 (0.14–0.89), GCA = 0.48 (0.26–0.89), and GNCA = 0.65 (0.45–0.94)] with significant tests for trends. In women, associations of aMEDr with GCA and GNCA risk were also inverse, but did not reach statistical significance [HR_per two−point increment_ (95% CI): GCA = 0.82 (0.51–1.33), GNCA = 0.83 (0.67–1.01)]. In contrast to men, aMEDr was not inversely associated with ESCC risk in women. EAC risk was not associated with aMEDr in both sexes. Heterogeneity tests showed that the association of aMEDr with ESCC risk differed significantly between the sexes (*p*_heterogeneity_ = 0.008). Associations of similar directions were observed when MD adherence was assessed using mMEDr in men, whereas we did not observe associations with any of the subtypes in women using mMEDr.

Overall, vegetable and fruit intakes were strong contributors to the inverse associations observed in male NLCS participants. High nut intake was associated with a significantly reduced GNCA risk (*p* = 0.008) in men, but did not contribute to the inverse association with ESCC risk. Furthermore, a low intake of red and processed meats contributed considerably to the inverse association with ESCC risk in men. Concerning the non-significant inverse associations with GCA and GNCA risk observed in women, intakes of nuts, whole grains, fish, and the MUFA:SFA ratio particularly contributed. In women, a high fish intake was associated with a significantly reduced GCA risk (*p* = 0.046) (data not shown).

Based on AIC values, models containing aMEDr performed similarly or better than mMEDr-containing models for all esophageal and gastric cancer subtypes considered. Inclusion of alcohol in aMED resulted in a clearly worse model fit when considering ESCC risk. Similar model performances were observed when aMED variants with and without alcohol were compared for EAC, GCA, and GNCA risks.

In the stratified analyses (Table 4), men and women were combined to increase the statistical power. Associations of aMEDr with all esophageal and gastric cancer subtypes were similar across strata of cigarette smoking status, alcohol consumption, and BMI. A significant interaction between aMEDr and level of education was observed for GNCA risk (*P*_heterogeneity_ = 0.0073) with a significant inverse association only being present in the lowest category. Inverse associations were also most apparent among those in the lowest education category when considering ESCC and GCA, but interaction tests were not significant for these subtypes. Finally, exclusion of the first 2 years of follow-up did not relevantly change the results (data not shown).

**Table 4:** Fully adjusted associations of aMEDr with the risk of esophageal and gastric cancer subtypes for various subgroups in the NLCS

	ESCC		EAC		GCA		GNCA	
	Cases	HR (95% CI)^ab^	Cases	HR (95% CI)^ab^	Cases	HR (95% CI)^ab^	Cases	HR (95% CI)^ab^
Overall	133	0.77 (0.61–0.98)	200	1.14 (0.94–1.37)	191	0.86 (0.71–1.04)	586	0.83 (0.73–0.93)
Cigarette smoking status^c^								
Never smoker	27	0.96 (0.54–1.68)	40	0.96 (0.64–1.44)	31	1.07 (0.64–1.80)	139	0.93 (0.74–1.17)
Former smoker	41	0.73 (0.47–1.12)	98	1.38 (1.04–1.84)	96	0.72 (0.55–0.96)	240	0.77 (0.64–0.93)
Current smoker	65	0.78 (0.55–1.10)	62	0.98 (0.68–1.39)	64	1.02 (0.73–1.42)	207	0.83 (0.67–1.03)
*P*_heterogeneity_^d^		0.7359		0.1553		0.3049		0.4399
Alcohol consumption^e^								
0 g/day	21	0.84 (0.44–1.60)	36	1.43 (0.87–2.33)	27	1.11 (0.66–1.85)	124	0.76 (0.57–0.99)
> 0–<15.0 g/day	47	1.05 (0.71–1.54)	90	0.99 (0.74–1.32)	97	0.83 (0.63–1.09)	296	0.90 (0.76–1.06)
≥ 15.0 g/day	65	0.60 (0.42–0.84)	74	1.33 (0.97–1.80)	67	0.75 (0.53–1.08)	166	0.73 (0.58–0.92)
*P*_heterogeneity_^d^		0.1293		0.1793		0.2517		0.3890
Body mass index^f^								
≥ 18.5–< 25.0 kg/m^2^	80	0.85 (0.62–1.15)	84	1.01 (0.75–1.37)	82	0.73 (0.55–0.96)	314	0.83 (0.71–0.98)
≥ 25.0 kg/m^2^	50	0.58 (0.38–0.90)	115	1.25 (0.98–1.61)	109	0.97 (0.74–1.27)	264	0.82 (0.68–0.98)
*P*_heterogeneity_^d^		0.3690		0.2248		0.1509		0.9198
Level of education^g^								
Primary school or lower vocational	66	0.54 (0.36–0.80)	103	0.94 (0.70–1.27)	96	0.71 (0.51–0.97)	356	0.72 (0.61–0.85)
Secondary school or medium vocational	49	1.00 (0.67–1.49)	63	1.28 (0.96–1.72)	56	0.89 (0.65–1.23)	169	0.89 (0.73–1.09)
Higher vocational or university	18	1.08 (0.68–1.72)	34	1.48 (0.87–2.54)	39	1.30 (0.86–1.97)	61	1.35 (0.93–1.95)
*P*_heterogeneity_^d^		0.1135		0.2762		0.0842		0.0073

*aMEDr* alternate Mediterranean diet score without the alcohol component, *NLCS* Netherlands Cohort Study, *ESCC* esophageal squamous cell carcinoma, *EAC* esophageal adenocarcinoma, *GCA* gastric cardia adenocarcinoma, *GNCA* gastric non-cardia adenocarcinoma, *HR* hazard ratio, *CI* confidence interval, *g*/*day* grams per day, *kg* kilogram, *m* meter

^a^All HRs were estimated per two-point increment in aMEDr

^b^Adjusted for age at baseline (years), sex (men, women), cigarette smoking status (never, former, current), cigarette smoking frequency (cigarettes smoked per day, centered), cigarette smoking duration (years, centered), body mass index (kg/m^2^), daily energy intake (kilocalories), alcohol consumption (g/day), highest level of education (primary school or lower vocational, secondary school or medium vocational, higher vocational or university), non-occupational physical activity (≤ 30, > 30-≤60, > 60-≤90, > 90 min per day), family history of esophageal cancer (for esophageal cancer subtypes; no, yes), and family history of gastric cancer (for gastric cancer subtypes; no, yes)

^c^Not adjusted for cigarette smoking status

^d^*P*-values for heterogeneity were obtained by testing the statistical significance of interaction terms between aMEDr and the stratifying covariates in fully adjusted models

^e^Not adjusted for alcohol consumption

^f^Not adjusted for body mass index

^g^Not adjusted for level of education

## Discussion

In the NLCS, higher aMEDr values were associated with significantly reduced risks of ESCC, GCA, and GNCA in men. In women, we observed non-significant inverse associations between aMEDr and risks of GCA and GNCA, but not ESCC. MD adherence was not associated with EAC risk. Associations of aMEDr with ESCC risk significantly differed between the sexes. Compared to mMEDr-containing models, aMEDr-containing models had similar or better performances. Model performances were generally comparable for aMED variants with and without alcohol, except for ESCC, where models containing aMED (including alcohol) clearly fitted worse.

Results of previously conducted prospective cohort studies [10, 11] investigating the association of *a priori* defined MD adherence with the risk of esophageal and/or gastric cancer subtypes were partially in accordance with our observations in the NLCS. In the US National Institutes of Health-American Association of Retired Persons Diet and Health study, high MD adherence (aMED) was associated with a significantly reduced risk of ESCC, but not EAC, GCA, and GNCA [11]. Although not statistically significant, the association with GNCA was also inverse. In the European Prospective Investigation into Cancer and Nutrition, inverse associations with MD adherence (relative Mediterranean diet score including alcohol, rMED) were suggested for total GC, GCA, and GNCA, but only reached statistical significance for total GC and GCA [10]. In accordance with the cohort evidence, an Italian case–control study also observed a significant inverse association between *a priori* defined MD adherence and ESCC risk [9]. Case–control studies focusing on gastric cancer risk found higher MD adherence (*a priori* defined) to be associated with significantly reduced risks of GCA, GNCA, and total GC [12, 13]. Finally, adherence to an *a posteriori* defined MD pattern was inversely associated with total GC risk in a Spanish case–control study [33]. Subtype-specific analyses showed that the inverse association was only statistically significant for GNCA. Combining male and female participants in the present study, HRs (95% CI) per two-point increment in aMEDr were 0.77 (0.61–0.98) for ESCC, 1.14 (0.94–1.37) for EAC, 0.86 (0.71–1.04) for GCA, and 0.83 (0.73–0.93) for GNCA.

For ESCC, associations with aMEDr significantly differed between male and female NLCS participants. Prior studies by Bosetti et al. [9] and Li et al. [11] did not observe a significant interaction between sex and MD adherence for ESCC risk. However, the inverse association in the latter study also seemed to be restricted to men [11]. Residual confounding by smoking behavior could potentially have caused the inverse association between aMEDr and ESCC risk that we observed in men. Tobacco smoking is strongly associated with an increased risk of ESCC [2, 34]. In our study, subjects in the highest aMEDr category were less likely to be current smokers. Since male participants were more likely to smoke than female participants, the effect of residual confounding by smoking behavior would be larger in men. This could potentially explain why we only observed an inverse association between aMEDr and ESCC risk in men. However, additional subgroup analyses for smoking status restricted to men showed that the inverse association between aMEDr and ESCC risk was strongest in men who had never smoked (data not shown), making it less likely that the observed differences between men and women in the NLCS were solely due to residual confounding by smoking behavior. Therefore, potential male–female differences in the association of MD adherence with ESCC risk deserve attention in future studies. For EAC, GCA, GNCA, and total GC, there was no evidence of heterogeneity between the sexes in neither the present study nor the literature [10–13].

In the present study, inverse associations between aMEDr and ESCC, GCA, and GNCA risk were most pronounced in subjects in the lowest education category with a significant interaction being observed for GNCA. Similarly, Li et al. [11] reported that aMED was only significantly inversely associated with EAC risk in the lowest education category (*p*_interaction_ = 0.02). However, there was no evidence of effect modification by educational level in the study by Praud et al. [12]. Although the interaction between aMEDr and educational level that we observed might be a chance finding due to the large number of tests performed, it should be investigated in future studies.

In their Third Expert Report, the World Cancer Research Fund/American Institute for Cancer Research suggested that high intakes of vegetables and fruits, and low intakes of processed meat and grilled (broiled) or barbecued (charbroiled) meat and fish might be associated with reduced risks of ESCC, EAC, and/or GC [26]. This is in correspondence with our observations that high intakes of vegetables, fruits and nuts, and a low intake of red and processed meats (ESCC only) particularly contributed to the inverse associations with ESCC, GCA, and GNCA risks in men in the NLCS. In women, nuts, whole grains, fish, and the MUFA:SFA ratio were important aMEDr components contributing to the non-significant inverse associations observed for GCA and GNCA risk. Inverse associations (not all significant) between esophageal and/or gastric cancer subtypes and intakes of nuts (ESCC and GNCA), vegetables (ESCC and EAC), and fruits (ESCC) were also documented in previous NLCS analyses [35, 36]. In addition, high intakes of red (non-significant) and processed meats were associated with an increased ESCC risk in men [37]. Although the above-mentioned aMEDr components were important contributors to the inverse associations that we observed in the present analysis, none of the individual components seemed to be the sole driver. This supports our pattern-based approach, which accounts for synergistic and antagonistic interactions between dietary components, and solves collinearity and confounding issues associated with the evaluation of individual components. Moreover, weak effects of single dietary components may only emerge when combined in dietary patterns [22, 38, 39].

Different etiologies have been suggested for the subtypes of esophageal and gastric cancer based on differences in risk factors and incidence trends [2, 3, 34, 40]. This stresses the importance of considering ESCC, EAC, GCA, and GNCA as separate outcomes as we did in this study. ESCC and EAC were clearly differently associated with MD adherence in our analysis. While MD adherence was significantly inversely associated with ESCC risk in men, no association was observed with EAC risk. In contrast to the esophageal cancer subtypes, GCA and GNCA seemed to have roughly comparable associations with MD adherence.

MD adherence might reduce cancer risk by decreasing oxidative stress, reactive oxygen species-induced DNA damage and inflammation [39, 41]. The MD is rich in antioxidants (e.g., vitamins and polyphenols) from plant foods and olive oil, and has been associated with higher total antioxidant capacity and lower levels of oxidized low-density lipoprotein cholesterol [26, 39, 41, 42]. Moreover, polyphenols (e.g., flavonoids) may reduce inflammation and MD adherence has been inversely associated with inflammatory biomarker concentrations [24, 43]. Finally, dietary fiber possibly acts as a nitrite scavenger, counteracting the carcinogenic effects of N-nitroso compounds [39, 44]. Low meat intake may also contribute to the cancer-protective effects of the MD. Nitrates and nitrites in processed meat can form N-nitroso compounds in the stomach. Besides, heme iron in red meat also stimulates the endogenous formation of N-nitroso compounds and causes oxidative stress and DNA damage. Finally, carcinogenic heterocyclic amines and polycyclic aromatic hydrocarbons are formed during high-temperature cooking of meat [26].

The use of data from a large prospective cohort with a long duration of follow-up enabled us to perform subtype- and sex-specific analyses. However, case numbers for the individual subtypes were low, necessitating us to combine men and women in the stratified and sensitivity analyses to increase the statistical power. Another strength of our study was the availability of high-quality dietary data. The NLCS-FFQ was validated using 9-day dietary records completed over three different seasons, showing an adequate performance [16]. Furthermore, a reproducibility study demonstrated that the single baseline measurement of this FFQ performed relatively well in ranking subjects according to their nutrient intake for over at least 5 years [19]. Nonetheless, changes in dietary habits and potential confounders during follow-up might still have attenuated the observed associations. Instead of using self-reporting based methods (e.g., FFQs and dietary records), dietary intake could be assessed by the measurement of biomarker concentrations in blood. However, there are currently no biomarkers available that assess adherence to all the aspects of the MD. Furthermore, biomarker levels in blood are also influenced by, e.g., absorption and excretion rates and reflect only short-term dietary intake.

Despite the fact that we adjusted for a large number of potential confounders, residual confounding by unmeasured factors may still exist. For example, we did not obtain data regarding *Helicobacter pylori* infection, which might have confounded our results in particular for GNCA. Furthermore, we cannot exclude the possibility of errors in the outcome measurements. However, it was reported that the histology and topography information from the Netherlands Cancer Registry, which we used to define the tumor subtypes, is of high accuracy [45]. Reversed causation due to the presence of preclinical disease symptoms in cases is another concern, particularly when investigating relations between dietary factors and gastrointestinal cancers, as we discussed previously [46]. Prospective cohort studies are less sensitive to this type of bias than case–control designs and we obtained similar results when excluding the first 2 years of follow-up.

Data on the reliability of *a priori* scores used in the assessment of MD adherence are limited [47]. The reliability of ten indexes measuring MD adherence, including mMED and rMED, has been evaluated by assessing correlations with a hidden common factor (obtained by factor analysis) understood as “MD adherence” [48]. Both mMED and rMED were amongst the four indexes that showed high correlations with the “MD adherence” factor (mMED: 0.83, rMED: 0.80) [48]. Regarding the validity of the MD scores used, subjects with higher MD score values in our study consumed, as expected, more plant foods (e.g., vegetables, legumes, fruits, and nuts) and less foods from animal origin (e.g., meat and dairy products). A similar pattern was previously observed for tMED [22]. However, the validity of tMED and its variants has mainly been established by showing inverse associations with various adverse health outcomes including all-cause mortality and risks of and mortality from cardiovascular diseases and cancer (e.g., [22, 23, 25, 49, 50]). Several studies have compared associations between various index-based dietary patterns, including tMED (variants), and cancer risk. A review published in 2018 showed that 3 out of 4 studies investigating the association of tMED (variants) with postmenopausal estrogen receptor-negative breast cancer risk observed a significant inverse relation. However, associations were inconsistent for other dietary pattern scores [e.g., Dietary Inflammatory Index (DII), Healthy Eating Index (HEI) and Alternate Healthy Eating Index (AHEI)] [51]. Considering prostate cancer risk, tMED (variants) and DII showed relatively consistent associations across studies, whereas the evidence was inconsistent and/or insufficient for the other indexes [52]. Furthermore, healthier diets according to tMED (variants), HEI/AHEI and DII have all been associated with lower risks of colorectal cancer [53]. More studies evaluating associations of various index-based dietary patterns with cancer risk are required to identify the preferred dietary pattern(s) in the perspective of cancer prevention. A final limitation of our study was the population-dependent assignment of scores in the assessment of MD adherence, which we have elaborately discussed previously [46].

In conclusion, high MD adherence was associated with reduced risks of ESCC, GCA, and GNCA in the NLCS. However, the inverse association with ESCC risk seemed to be restricted to men. So far, results for esophageal cancer generally were consistent with high MD adherence being associated with a reduced risk of ESCC, but not EAC. Findings concerning GCA and GNCA were more diverse, but, generally, inverse associations (not always significant) were observed for at least one of the subtypes. The potential differences in associations between men and women, particularly for ESCC, require further attention.

## Electronic supplementary material

Below is the link to the electronic supplementary material.

Supplementary material 1 (PDF 533 KB)

## References

[1] Ferlay J, Soerjomataram I, Ervik M, Dikshit R, Eser S, Mathers C, et al. GLOBOCAN 2012 v1.0, Cancer Incidence and Mortality Worldwide: IARC CancerBase No. 11 [Internet]. Lyon, France: International Agency for Research on Cancer; 2013 [cited 2018 July]. Available from: http://globocan.iarc.fr.

[2] Kamangar F, Chow WH, Abnet CC, Dawsey SM (2009). Environmental causes of esophageal cancer. Gastroenterol Clin North Am.

[3] Crew KD, Neugut AI (2006). Epidemiology of gastric cancer. World J Gastroenterol.

[4] Brown LM, Devesa SS (2002). Epidemiologic trends in esophageal and gastric cancer in the United States. Surg Oncol Clin N Am.

[5] Steevens J, Botterweck AA, Dirx MJ, van den Brandt PA, Schouten LJ (2010). Trends in incidence of oesophageal and stomach cancer subtypes in Europe. Eur J Gastroenterol Hepatol.

[6] Willett WC, Sacks F, Trichopoulou A, Drescher G, Ferro-Luzzi A, Helsing E (1995). Mediterranean diet pyramid: a cultural model for healthy eating. Am J Clin Nutr.

[7] Trichopoulou A, Lagiou P (1997). Healthy traditional Mediterranean diet: an expression of culture, history, and lifestyle. Nutr Rev.

[8] Fung TT, Rexrode KM, Mantzoros CS, Manson JE, Willett WC, Hu FB (2009). Mediterranean diet and incidence of and mortality from coronary heart disease and stroke in women. Circulation.

[9] Bosetti C, Gallus S, Trichopoulou A, Talamini R, Franceschi S, Negri E (2003). Influence of the Mediterranean diet on the risk of cancers of the upper aerodigestive tract. Cancer Epidemiol Biomarkers Prev.

[10] Buckland G, Agudo A, Lujan L, Jakszyn P, Bueno-de-Mesquita HB, Palli D (2010). Adherence to a Mediterranean diet and risk of gastric adenocarcinoma within the European Prospective Investigation into Cancer and Nutrition (EPIC) cohort study. Am J Clin Nutr.

[11] Li WQ, Park Y, Wu JW, Ren JS, Goldstein AM, Taylor PR (2013). Index-based dietary patterns and risk of esophageal and gastric cancer in a large cohort study. Clin Gastroenterol Hepatol.

[12] Praud D, Bertuccio P, Bosetti C, Turati F, Ferraroni M, La Vecchia C (2014). Adherence to the Mediterranean diet and gastric cancer risk in Italy. Int J Cancer.

[13] Stojanovic J, Giraldi L, Arzani D, Pastorino R, Biondi A, Persiani R (2017). Adherence to Mediterranean diet and risk of gastric cancer: results of a case–control study in Italy. Eur J Cancer Prev.

[14] van den Brandt PA, Goldbohm RA, van’t Veer P, Volovics A, Hermus RJ, Sturmans F (1990). A large-scale prospective cohort study on diet and cancer in The Netherlands. J Clin Epidemiol.

[15] van den Brandt PA, Schouten LJ, Goldbohm RA, Dorant E, Hunen PM (1990). Development of a record linkage protocol for use in the Dutch Cancer Registry for Epidemiological Research. Int J Epidemiol.

[16] Goldbohm RA, van den Brandt PA, Brants HA, van’t Veer P, Al M, Sturmans F (1994). Validation of a dietary questionnaire used in a large-scale prospective cohort study on diet and cancer. Eur J Clin Nutr.

[17] Volovics A, van den Brandt PA (1997). Methods for the analyses of case–cohort studies. Biometrical J.

[18] Prentice RL (1986). A case-cohort design for epidemiologic cohort studies and disease prevention trials. Biometrika.

[19] Goldbohm RA, van ’t Veer P, van den Brandt PA, van ’t Hof MA, Brants HA, Sturmans F (1995). Reproducibility of a food frequency questionnaire and stability of dietary habits determined from five annually repeated measurements. Eur J Clin Nutr.

[20] NEVO, table (1986). Dutch food composition table 1986–1987.

[21] Trichopoulou A, Kouris-Blazos A, Wahlqvist ML, Gnardellis C, Lagiou P, Polychronopoulos E (1995). Diet and overall survival in elderly people. BMJ.

[22] Trichopoulou A, Costacou T, Bamia C, Trichopoulos D (2003). Adherence to a Mediterranean diet and survival in a Greek population. N Engl J Med.

[23] Trichopoulou A, Orfanos P, Norat T, Bueno-de-Mesquita B, Ocke MC, Peeters PH (2005). Modified Mediterranean diet and survival: EPIC-elderly prospective cohort study. BMJ.

[24] Fung TT, McCullough ML, Newby PK, Manson JE, Meigs JB, Rifai N (2005). Diet-quality scores and plasma concentrations of markers of inflammation and endothelial dysfunction. Am J Clin Nutr.

[25] Mitrou PN, Kipnis V, Thiebaut AC, Reedy J, Subar AF, Wirfalt E (2007). Mediterranean dietary pattern and prediction of all-cause mortality in a US population: results from the NIH-AARP Diet and Health Study. Arch Intern Med.

[26] World Cancer Research Fund/American Institute for Cancer Research. Diet, nutrition, physical activity and cancer: a global perspective. Continuous update project expert report 2018. 2018. Available from: https://www.wcrf.org/dietandcancer.

[27] Lin DY, Wei LJ (1989). The robust inference for the cox proportional hazards model. J Am Stat Assoc.

[28] Grambsch PM, Therneau TM (1994). Proportional hazards tests and diagnostics based on weighted residuals. Biometrika.

[29] Akaike H (1974). A new look at the statistical model identification. IEEE Trans Autom Control.

[30] van den Brandt PA, Schulpen M (2017). Mediterranean diet adherence and risk of postmenopausal breast cancer: results of a cohort study and meta-analysis. Int J Cancer.

[31] Schulpen M, van den Brandt PA (2018). Adherence to the Mediterranean diet and risk of lung cancer in the Netherlands Cohort Study. Br J Nutr.

[32] Trichopoulou A, Bamia C, Trichopoulos D (2009). Anatomy of health effects of Mediterranean diet: Greek EPIC prospective cohort study. BMJ.

[33] Castello A, Fernandez de Larrea N, Martin V, Davila-Batista V, Boldo E, Guevara M (2018). High adherence to the Western, Prudent, and Mediterranean dietary patterns and risk of gastric adenocarcinoma: MCC-Spain study. Gastric Cancer.

[34] Domper Arnal MJ, Ferrandez Arenas A, Lanas Arbeloa A (2015). Esophageal cancer: risk factors, screening and endoscopic treatment in Western and Eastern countries. World J Gastroenterol.

[35] Steevens J, Schouten LJ, Goldbohm RA, van den Brandt PA (2011). Vegetables and fruits consumption and risk of esophageal and gastric cancer subtypes in the Netherlands Cohort Study. Int J Cancer.

[36] Nieuwenhuis L, van den Brandt PA (2018). Tree nut, peanut, and peanut butter consumption and the risk of gastric and esophageal cancer subtypes: the Netherlands Cohort Study. Gastric Cancer.

[37] Keszei AP, Schouten LJ, Goldbohm RA, van den Brandt PA (2012). Red and processed meat consumption and the risk of esophageal and gastric cancer subtypes in The Netherlands Cohort Study. Ann Oncol.

[38] Jacques PF, Tucker KL (2001). Are dietary patterns useful for understanding the role of diet in chronic disease?. Am J Clin Nutr.

[39] Verberne L, Bach-Faig A, Buckland G, Serra-Majem L (2010). Association between the Mediterranean diet and cancer risk: a review of observational studies. Nutr Cancer.

[40] Jemal A, Center MM, DeSantis C, Ward EM (2010). Global patterns of cancer incidence and mortality rates and trends. Cancer Epidemiol Biomark Prev.

[41] Brill JB (2009). The Mediterranean diet and your health. Am J Lifestyle Med.

[42] Pitsavos C, Panagiotakos DB, Tzima N, Chrysohoou C, Economou M, Zampelas A (2005). Adherence to the Mediterranean diet is associated with total antioxidant capacity in healthy adults: the ATTICA study. Am J Clin Nutr.

[43] Rahman I, Biswas SK, Kirkham PA (2006). Regulation of inflammation and redox signaling by dietary polyphenols. Biochem Pharmacol.

[44] Moller ME, Dahl R, Bockman OC (1988). A possible role of the dietary fibre product, wheat bran, as a nitrite scavenger. Food Chem Toxicol.

[45] Schouten LJ, Jager JJ, van den Brandt PA (1993). Quality of cancer registry data: a comparison of data provided by clinicians with those of registration personnel. Br J Cancer.

[46] Schulpen M, Peeters PH, van den Brandt PA (2018). Mediterranean diet adherence and risk of pancreatic cancer: a pooled analysis of two Dutch cohorts. Int J Cancer.

[47] Zaragoza-Marti A, Cabanero-Martinez MJ, Hurtado-Sanchez JA, Laguna-Perez A, Ferrer-Cascales R (2018). Evaluation of Mediterranean diet adherence scores: a systematic review. BMJ Open.

[48] Mila-Villarroel R, Bach-Faig A, Puig J, Puchal A, Farran A, Serra-Majem L (2011). Comparison and evaluation of the reliability of indexes of adherence to the Mediterranean diet. Public Health Nutr.

[49] Couto E, Boffetta P, Lagiou P, Ferrari P, Buckland G, Overvad K (2011). Mediterranean dietary pattern and cancer risk in the EPIC cohort. Br J Cancer.

[50] Buckland G, Gonzalez CA, Agudo A, Vilardell M, Berenguer A, Amiano P (2009). Adherence to the Mediterranean diet and risk of coronary heart disease in the Spanish EPIC Cohort Study. Am J Epidemiol.

[51] Du M, Liu SH, Mitchell C, Fung TT (2018). Associations between diet quality scores and risk of postmenopausal estrogen receptor-negative breast cancer: a systematic review. J Nutr.

[52] Kim JH, Kim J (2017). Index-based dietary patterns and the risk of prostate cancer. Clin Nutr Res.

[53] Steck SE, Guinter M, Zheng J, Thomson CA (2015). Index-based dietary patterns and colorectal cancer risk: a systematic review. Adv Nutr.

