# Supplementary material for: Mediterranean diet adherence and risk of esophageal and gastric cancer subtypes in the Netherlands Cohort Study
Source: Gastric Cancer. 2019 Feb 15;22(4):663–74. doi: 10.1007/s10120-019-00927-x (PMC6570688; doi:10.1007/s10120-019-00927-x)
Supplement: Supplementary file 1 — Supplementary material 1 (PDF 533 KB) [file 10120_2019_927_MOESM1_ESM.pdf]

Mediterranean diet adherence and risk of esophageal and gastric cancer subtypes in the Netherlands Cohort Study

Gastric Cancer

Maya Schulpen, Petra H. Peeters, Piet A. van den Brandt

Corresponding author: Piet A. van den Brandt, Maastricht University Medical Centre, Department of Epidemiology, P.O. Box 616, 6200 MD Maastricht, the Netherlands. Phone: +31 (0)43 3882361, Fax: +31 (0)43 3884128, E-mail: pa.vandenbrandt@maastrichtuniversity.nl

Online Resource 1 Age-adjusted associations of aMEDr and mMEDr with the risk of esophageal and gastric cancer subtypes in male and female NLCS participants

			ESCC		EAC		GCA		GNCA	
		$PY_{subcohort}$	Cases	HR (95% CI)[a]	Cases	HR (95% CI)[a]	Cases	HR (95% CI)[a]	Cases	HR (95% CI)[a]
Men	aMEDr									
	0-3	11889	46	1.00	59	1.00	71	1.00	190	1.00
	4-5	12569	25	0.52 (0.31 - 0.85)	70	1.12 (0.78 - 1.61)	74	0.99 (0.70 - 1.39)	155	0.78 (0.61 - 0.99)
	6-8	4792	5	0.26 (0.10 - 0.67)	28	1.14 (0.71 - 1.83)	13	0.45 (0.24 - 0.82)	45	0.57 (0.40 - 0.81)
	P_{trend}			0.002		0.575		0.008		0.001
	Continuous, per 2 pts	29250	76	0.54 (0.40 - 0.72)	157	1.10 (0.89 - 1.35)	158	0.83 (0.68 - 1.02)	390	0.79 (0.69 - 0.90)
	mMEDr									
	0-3	10920	39	1.00	61	1.00	66	1.00	156	1.00
	4-5	13549	29	0.58 (0.36 - 0.96)	68	0.88 (0.61 - 1.27)	75	0.90 (0.64 - 1.28)	182	0.91 (0.71 - 1.16)
	6-8	4782	8	0.45 (0.21 - 0.99)	28	1.03 (0.64 - 1.65)	17	0.58 (0.33 - 1.01)	52	0.73 (0.52 - 1.03)
	P_{trend}			0.039		0.930		0.049		0.075
	Continuous, per 2 pts	29250	76	0.65 (0.49 - 0.85)	157	0.99 (0.79 - 1.24)	158	0.82 (0.66 - 1.01)	390	0.91 (0.79 - 1.05)
Women	aMEDr									
	0-3	12254	21	1.00	19	1.00	15	1.00	92	1.00
	4-5	15123	27	1.07 (0.60 - 1.93)	18	0.78 (0.40 - 1.50)	12	0.65 (0.30 - 1.39)	71	0.64 (0.46 - 0.89)
	6-8	6278	9	0.89 (0.40 - 1.99)	6	0.64 (0.25 - 1.62)	6	0.80 (0.31 - 2.04)	33	0.74 (0.48 - 1.13)
	P_{trend}			0.789		0.340		0.638		0.153
	Continuous, per 2 pts	33655	57	0.98 (0.70 - 1.36)	43	0.94 (0.65 - 1.36)	33	0.81 (0.50 - 1.31)	196	0.77 (0.63 - 0.94)
	mMEDr									
	0-3	11675	17	1.00	17	1.00	9	1.00	75	1.00
	4-5	16510	31	1.32 (0.72 - 2.41)	19	0.80 (0.41 - 1.56)	18	1.42 (0.64 - 3.19)	91	0.88 (0.63 - 1.21)
	6-8	5470	9	1.19 (0.52 - 2.72)	7	0.90 (0.37 - 2.21)	6	1.45 (0.51 - 4.09)	30	0.89 (0.57 - 1.40)
	P_{trend}			0.659		0.812		0.477		0.597
	Continuous, per 2 pts	33655	57	1.17 (0.81 - 1.67)	43	0.92 (0.62 - 1.37)	33	1.03 (0.67 - 1.58)	196	0.95 (0.78 - 1.16)

aMEDr alternate Mediterranean diet score without the alcohol component, *mMEDr* modified Mediterranean diet score without the alcohol component, *NLCS* Netherlands Cohort Study, *ESCC* esophageal squamous cell carcinoma, *EAC* esophageal adenocarcinoma, *GCA* gastric cardia adenocarcinoma, *GNCA* gastric non-cardia adenocarcinoma, $PY_{subcohort}$ person-years in the subcohort, *HR* hazard ratio, *CI* confidence interval, *pts* points

[a] Adjusted for age at baseline (years).
